# Genomic Surveillance of Enterococcus faecium Reveals Limited Sharing of Strains and Resistance Genes between Livestock and Humans in the United Kingdom

**DOI:** 10.1128/mBio.01780-18

**Published:** 2018-11-06

**Authors:** Theodore Gouliouris, Kathy E. Raven, Catherine Ludden, Beth Blane, Jukka Corander, Carolyne S. Horner, Juan Hernandez-Garcia, Paul Wood, Nazreen F. Hadjirin, Milorad Radakovic, Mark A. Holmes, Marcus de Goffau, Nicholas M. Brown, Julian Parkhill, Sharon J. Peacock

**Affiliations:** a Department of Medicine, University of Cambridge, Cambridge, United Kingdom; b Public Health England, Clinical Microbiology and Public Health Laboratory, Cambridge University Hospitals NHS Foundation Trust, Cambridge, United Kingdom; c London School of Hygiene and Tropical Medicine, London, United Kingdom; d Department of Biostatistics, Institute of Basic Medical Sciences, Faculty of Medicine, University of Oslo, Oslo, Norway; e Wellcome Sanger Institute, Cambridge, United Kingdom; f British Society for Antimicrobial Chemotherapy, Birmingham, United Kingdom; g Department of Veterinary Medicine, University of Cambridge, Cambridge, United Kingdom; h Royal (Dick) School of Veterinary Studies, University of Edinburgh, Edinburgh, United Kingdom; University of Birmingham; Emory University School of Medicine

**Keywords:** vancomycin-resistant, *Enterococcus faecium*, One Health, livestock, genome sequencing, vancomycin resistant

## Abstract

Vancomycin-resistant Enterococcus faecium (VREfm) is a major cause of nosocomial infection and is categorized as high priority by the World Health Organization global priority list of antibiotic-resistant bacteria. In the past, livestock have been proposed as a putative reservoir for drug-resistant E. faecium strains that infect humans, and isolates of the same lineage have been found in both reservoirs. We undertook cross-sectional surveys to isolate E. faecium (including VREfm) from livestock farms, retail meat, and wastewater treatment plants in the United Kingdom. More than 600 isolates from these sources were sequenced, and their relatedness and antibiotic resistance genes were compared with genomes of almost 800 E. faecium isolates from patients with bloodstream infection in the United Kingdom and Ireland. E. faecium was isolated from 28/29 farms; none of these isolates were VREfm, suggesting a decrease in VREfm prevalence since the last UK livestock survey in 2003. However, VREfm was isolated from 1% to 2% of retail meat products and was ubiquitous in wastewater treatment plants. Phylogenetic comparison demonstrated that the majority of human and livestock-related isolates were genetically distinct, although pig isolates from three farms were more genetically related to human isolates from 2001 to 2004 (minimum of 50 single-nucleotide polymorphisms [SNPs]). Analysis of accessory (variable) genes added further evidence for distinct niche adaptation. An analysis of acquired antibiotic resistance genes and their variants revealed limited sharing between humans and livestock. Our findings indicate that the majority of E. faecium strains infecting patients are largely distinct from those from livestock in this setting, with limited sharing of strains and resistance genes.

## Abstract (precis)

The rise in rates of human infection caused by vancomycin-resistant Enterococcus faecium (VREfm) strains between 1988 to the 2000s in Europe was suggested to be associated with acquisition from livestock. As a result, the European Union banned the use of the glycopeptide drug avoparcin as a growth promoter in livestock feed. While some studies reported a decrease in VREfm in livestock, others reported no reduction. Here, we report the first livestock VREfm prevalence survey in the UK since 2003 and the first large-scale study using whole-genome sequencing to investigate the relationship between E. faecium strains in livestock and humans. We found a low prevalence of VREfm in retail meat and limited evidence for recent sharing of strains between livestock and humans with bloodstream infection. There was evidence for limited sharing of genes encoding antibiotic resistance between these reservoirs, a finding which requires further research.

## INTRODUCTION

Enterococcus faecium is a major cause of nosocomial infection in immunocompromised and critically ill patients (1) and is a globally distributed cause of disease (2). Vancomycin-resistant E. faecium (VREfm) was first reported in 1988 (3, 4), followed during the 1990s by a rapid increase in rates of VREfm infections in hospitals in the United Kingdom (5). It was proposed that humans may have acquired VREfm or vancomycin resistance genes from livestock, linked to the use in Europe of avoparcin (an analog of vancomycin) as a growth promoter (6). Avoparcin was subsequently banned in Europe in 1997, after which many studies reported a decrease in VREfm in livestock (7). However, VREfm never completely disappeared from livestock (8), and one study even reported increased VREfm prevalence (9). The last study of VREfm in livestock to be conducted in the United Kingdom was in 2003, and it reported the isolation of VREfm in 27/33 poultry and 4/14 pig farms (10). Despite the ban on avoparcin use, rates of VREfm infections in humans have remained high, and the World Health Organization (WHO) global priority list of antibiotic-resistant bacteria has recently categorized VREfm as high priority (11). This reflects limited treatment options and higher mortality and health care costs for infections by vancomycin-resistant versus vancomycin-susceptible enterococci (12–16).

The failure to control VREfm infections in the United Kingdom is in stark contrast to the success with methicillin-resistant Staphylococcus aureus and Clostridium difficile infections (17). Preventing infection depends on defining and controlling the sources from which bacteria are acquired. While patients and the hospital environment have been implicated as important sources of VREfm infections (18, 19), strategies to more generally tackle antibiotic resistance have highlighted the need to identify and contain additional reservoirs of antimicrobial resistance (20). Whole-genome sequencing has been shown to be a highly discriminatory technique for E. faecium and other bacterial species. However, to date, only a single whole-genome sequencing study limited to 10 European livestock isolates has compared E. faecium isolates from livestock to those from other reservoirs (21). This landmark genomic study found that isolates from livestock and human infections formed distinct clades, confirming earlier, larger-scale studies that used more limited typing schemes (22). Further sequencing of hundreds of isolates from human infections has confirmed that these isolates predominantly belong to a hospital-associated clade (18, 23). However, human and livestock isolates belonging to the same sequence types (STs) are well described (24, 25), although detailed genomic studies to define relatedness between human and livestock isolates in the same lineage are lacking.

Here, we present the findings of the first large-scale whole-genome sequencing study of E. faecium from livestock and clinical isolates and the most comprehensive analysis to date of E. faecium from different reservoirs. We focus on two categories of analyses, first comparing the core genomes of E. faecium isolates from different reservoirs to determine phylogenetic relatedness and second, defining the extent to which genes and genetic elements in the accessory genome (including those encoding drug resistance) were shared.

## RESULTS

### Isolation of E. faecium from farms and retail meat.

We isolated E. faecium from livestock during a cross-sectional survey of 29 farms (ten cattle, ten pig, and nine poultry) in the East of England (UK), between 2014 and 2015 (Fig. S1A). We cultured 142 samples (136 pooled fecal/cecal samples [35 from cattle, 51 from pigs, and 50 from poultry], 2 porcine cecal samples from necropsy, and 4 fresh farm runoffs) for E. faecium on culture media that was selective for enterococci, ampicillin-resistant enterococci (a surrogate marker for hospital-associated E. faecium lineages; see reference 26), or VREfm after enrichment. Ampicillin-susceptible and -resistant E. faecium isolates were isolated from 28 and 19 farms, respectively, but no samples grew VREfm. Despite these negative results, people could still be exposed to VREfm in meat produced elsewhere. To investigate this, we undertook two independent cross-sectional surveys of prepackaged fresh meat. The first tested 103 products labeled as UK farm origin from 28 high-street supermarkets from five regions of the United Kingdom in February to March 2015. The second tested 97 products purchased in 11 high-street supermarkets in Cambridge, East of England, in April 2015. These originated from multiple countries although the majority (69/97; 71%) were from the United Kingdom (Table S1). Three chicken products from the United Kingdom were positive for VREfm. Two (2%) were from the United Kingdom-wide survey, and one was (1%) from the Cambridge survey (corresponding to 3.7% of all chicken products), and they originated from two supermarket chains in three different store locations.

10.1128/mBio.01780-18.1 FIG S1 Farm characteristics and geographical and phylogenetic distribution of *E. faecium* isolates cultured from livestock. Download FIG S1, PDF file, 0.3 MB. Copyright © 2018 Gouliouris et al. 2018 Gouliouris et al. This content is distributed under the terms of the Creative Commons Attribution 4.0 International license.

10.1128/mBio.01780-18.3 TABLE S1 Meat survey sample details. Download Table S1, XLSX file, 0.01 MB. Copyright © 2018 Gouliouris et al. 2018 Gouliouris et al. This content is distributed under the terms of the Creative Commons Attribution 4.0 International license.

### Phylogenetic description of E. faecium from farms and retail meat.

We sequenced the three VREfm meat isolates and 253 E. faecium isolates from livestock (52% ampicillin resistant), from which 94 multilocus sequence types (STs) were derived, including 35 novel STs (Table S2). The three meat isolates all belonged to the same novel ST, sequence type 1249 (ST1249), which was not represented in our livestock collection. A maximum-likelihood phylogeny based on single-nucleotide polymorphisms (SNPs) in the core genes of the 256 isolates demonstrated two distinct lineages, containing 249 (97%) and seven (3%) isolates, respectively (Fig. S1B). E. faecium isolates from each livestock species were genetically diverse and distributed across the phylogeny. Clustering was observed for isolates from the same species, together with relatedness of isolates from different species (particularly between chickens and turkeys and between pigs and cattle). The three meat isolates resided on their own branch in the predominant lineage and differed from each other by 27 to 61 SNPs, compared with a SNP difference of 2,390 from the closest livestock isolate (from a turkey).

10.1128/mBio.01780-18.4 TABLE S2 Isolate details. Download Table S2, XLSX file, 0.1 MB. Copyright © 2018 Gouliouris et al. 2018 Gouliouris et al. This content is distributed under the terms of the Creative Commons Attribution 4.0 International license.

### Phylogenetic relatedness of E. faecium isolated from patients with bloodstream infection and nonhuman sources.

**FIG 1:**
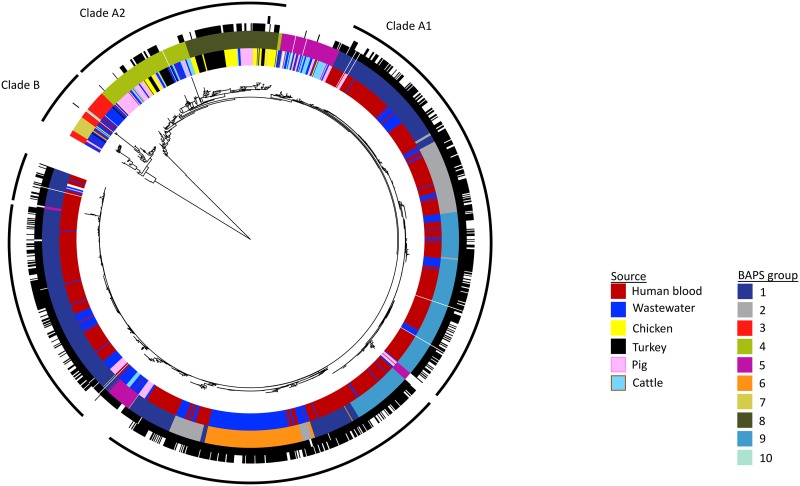
Maximum likelihood tree based on SNPs in the genes core to the 1,442 *E. faecium* isolates in the collection. Colored rings from inner to outer indicate isolate source, BAPS group, phenotypic resistance to ampicillin (black, resistant; white, susceptible/intermediate), and phenotypic resistance to vancomycin (black, resistant; white, susceptible). Outermost ring indicates the clades, with the gaps in Clade A1 corresponding to BAPS5.

We compared the genetic relatedness of the 256 livestock/meat E. faecium genomes with 782 E. faecium genomes associated with bloodstream infections in patients in the United Kingdom (362 from hospitals in the East of England), 383 E. faecium isolates isolated from 20 municipal wastewater treatment plants (Fig. S1A) in the East of England, 11 NCTC strains, and 10 other reference strains. A maximum-likelihood phylogenetic tree of the 1,442 isolate genomes based on core gene SNPs confirmed previous observations that the E. faecium population is divided into two major lineages (referred to previously as clade A (comprising hospital-associated A1 and animal-associated A2) and clade B (community-associated); see reference 21) (Fig. 1). The population structure was further categorized using Bayesian analysis, which identified ten Bayesian analysis of population structure (BAPS) groups (Fig. 1). Of these, BAPS3 (*n* = 39 isolates), BAPS7 (*n* = 18), and BAPS10 (*n* = 3) resided in clade B; BAPS1 (*n* = 529), BAPS2 (*n* = 138), BAPS6 (*n* = 117), and BAPS9 (*n* = 262) in clade A1; BAPS4 (*n* = 117) and BAPS8 (*n* = 108) in clade A2; and BAPS5 (*n* = 111) was intermediate between clade A1 and A2. BAPS groups were paraphyletic (located in two different positions on the tree), particularly BAPS5 and the clade A1 BAPS groups, which is likely to be due to the highly dynamic nature of the E. faecium genome and recombination between different lineages. There was notable segregation of BAPS groups associated with livestock (e.g., BAPS4 and BAPS8; 75% livestock) versus human invasive isolates (e.g., BAPS1, BAPS2, BAPS6, and BAPS9; 96% human invasive isolates) despite selecting for ampicillin-resistant livestock isolates, which we predicted would enrich for hospital-associated lineages. In contrast, we noted extensive mixing between isolates from wastewater and human disease and between those from wastewater and livestock (Fig. 1). The wastewater isolates originated from multiple treatment plants in all three clades, with all 20 treatment plants represented in clade A1. Of note, one BAPS group in clade A1 (BAPS6) contained predominantly wastewater isolates (91%, mostly ST80 [84%]), which were collected from 17 different wastewater treatment plants. This may either be a dominant lineage in the environment or, more likely, may represent a recent emergence of a lineage. The three VREfm meat isolates belonged to BAPS8 and resided within a poultry lineage.

Next, we defined the degree of genetic relatedness between 1,144 deduplicated livestock, human, and wastewater genomes in a pairwise comparison of SNPs after mapping to BAPS group-specific references. Deduplication was performed by removing isolates within 0 to 2 SNPs of each other from the same farm, wastewater plant, or hospital. Within the animal-associated BAPS groups (BAPS4 and BAPS8) and BAPS5, the most closely related isolate pair from livestock and humans differed by 129 SNPs, which, given a mutation rate of 7 SNPs/genome/year (18), equates to approximately 18 years of evolution. However, the most closely related isolate pair in the hospital-associated BAPS groups (comprising BAPS1, BAPS2, BAPS6, and BAPS9) was 40 SNPs apart. Based on the time between isolation and an estimated mutation rate of 7 SNPs/genome/year, 6 of the 7 pig isolates (from three different farms) residing in clade A1 after deduplication were related to 17 human isolates (50 to 91 SNPs different, with 10 to 13 years between sampling) from across the United Kingdom and Northern Ireland isolated between 2001 and 2004. No isolates from cattle, chickens, or turkeys were closely related to the human isolates. The six pig isolates represented 10% of the deduplicated pig collection (6/60), but 5/6 of these pig isolates were ampicillin resistant and thus had been enriched for by the selection process. We also identified closely related isolate pairs from livestock and wastewater and from humans and wastewater (minimum 8- and 2-SNP difference, respectively) suggesting sharing within approximately the last year between these reservoirs.

### Genetic relatedness of E. faecium isolates within and between livestock species.

**FIG 2:**
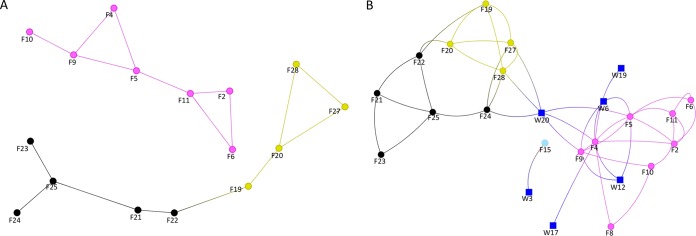
Network analysis of *E. faecium* isolates from farms and wastewater treatment plants. Lines are drawn between farms/plants sharing isolates less than seven SNPs (A) or 35 SNPs (B) apart, indicating approximately 1 and 5 years of evolution, respectively. Lines only show binary links between farms, with arbitrary line lengths. Blue square, wastewater treatment plant; pink circle, pig farm; light blue circle, cattle farm; black circle, turkey farm; yellow circle, chicken farm.

To address the relatedness of E. faecium strains within and between livestock species and farms, a network was created based on a pairwise comparison, in which pairs of livestock isolates were classified as genetically related if they were less than or equal to seven SNPs different (equivalent to approximately 1 year of evolution). There was no evidence of E. faecium relatedness between different livestock species, with the exception of E. faecium relatedness between chickens and turkeys (Fig. 2A). In contrast, there was evidence for relatedness of E. faecium strains within livestock species (chickens, turkeys, and pigs, but not cattle) across different farms. Reanalysis using a 35-SNP cutoff (an estimated 5 years since most recent common ancestor) extended these links and identified links between farms and wastewater isolates (Fig. 2B). There was no evidence for sharing between cattle or pigs versus other livestock species using this cutoff, with minimums of 477 and 292 SNPs identified, respectively.

### Comparison of accessory gene content of E. faecium isolates from human and nonhuman sources.

**FIG 3:**
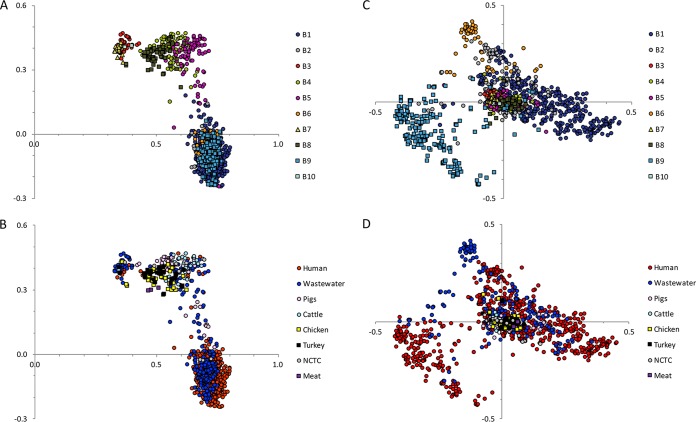
Principal component analysis based on the presence/absence of *E. faecium accessory* genes across the study collection. Panels A and B show principal component (PC) 1 (*x* axis) against PC2 (*y* axis) labeled by BAPS group (A) or isolate source (B). Panels C and D show PC3 (*x* axis) against PC4 (*y* axis) labeled by BAPS group (C) or isolate source (D). Principal components 1, 2, 3, and 4 explain 46%, 5%, 3%, and 2% of the variation within the accessory genome, respectively.

We then evaluated and compared the variable (accessory) genome of the 1,144 deduplicated study isolates. On principal component analysis, principal component 1 (PC1) and PC2 predominantly segregated the accessory genome into the three clades (A1, A2, and B), with BAPS5 clustering with A2 (Fig. 3A and B). Twenty-five isolates were positioned between the animal and hospital-associated clades, suggesting sharing of accessory genes. Similar to the core gene phylogeny, 12 human isolates clustered with clade A2, six livestock with clade A1, and 12 human and nine livestock isolates with clade B. Wastewater isolates were distributed throughout the plot, consistent with this being a representation of both livestock and human lineages. PC3 and PC4 predominantly segregated BAPS9 and BAPS6 (Fig. 3C and D), indicating lineage-specific traits within the hospital-associated clade. Genes associated with BAPS9 included phage-related genes, while genes associated with BAPS6 included transposase and phosphotransferase system (PTS) genes (Table S3), the latter being a recognized marker of adaptation (21).

10.1128/mBio.01780-18.5 TABLE S3 Genes associated with BAPS6 or BAPS9 based on principal component analysis of accessory genes. Download Table S3, XLSX file, 0.03 MB. Copyright © 2018 Gouliouris et al. 2018 Gouliouris et al. This content is distributed under the terms of the Creative Commons Attribution 4.0 International license.

**FIG 4:**
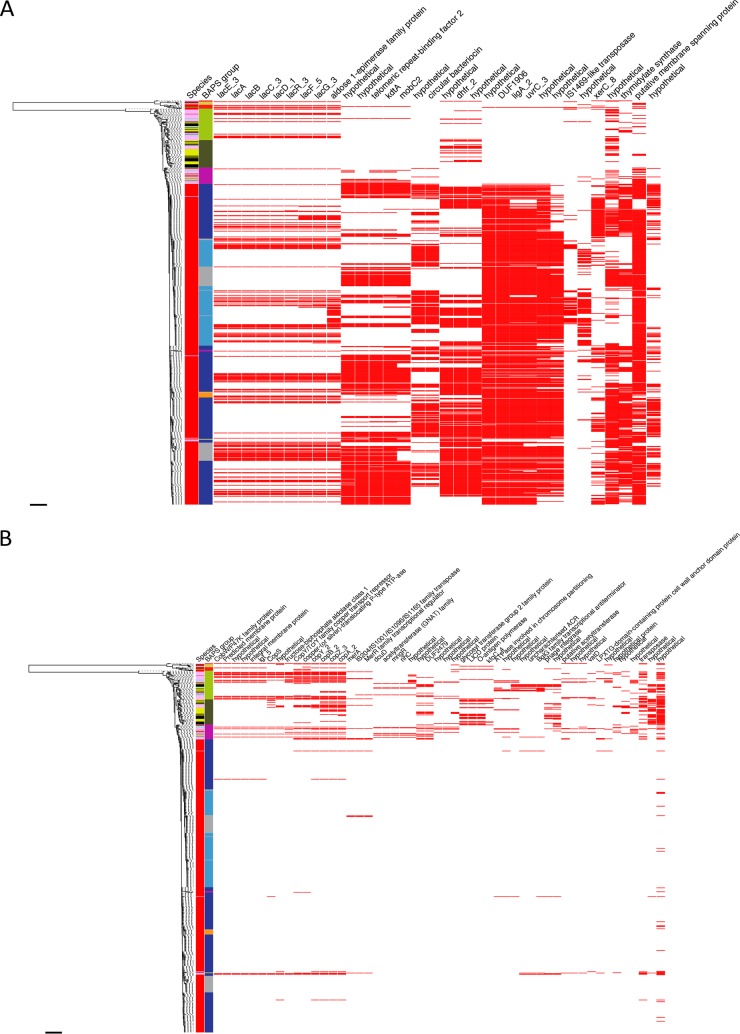
Genome-wide association study to detect genes that were overrepresented in *E. faecium* isolates from livestock and humans. (Left) maximum likelihood tree based on SNPs in the core genes of farm animal and human invasive isolates (*n* = 849). First two columns show the source species (red, human; pink, pig; light blue, cattle; black, turkey; yellow, chicken) and BAPS group (dark blue, BAPS1; gray, BAPS2; red, BAPS3; light green, BAPS4; pink, BAPS5; orange, BAPS6; yellow, BAPS7; dark green, BAPS8; light blue, BAPS9). Further columns are based on the accessory genome and show the presence (red) or absence (white) of genes strongly associated with humans (A) or animals (B). Columns are ordered based on the pattern of presence and absence. Scale bars indicate ∼30,000 SNPs.

We also performed a genome-wide association study on the 849 deduplicated livestock and human genomes to detect accessory genes that were significantly associated with livestock or human isolates. Thirty-two genes were significantly associated with human isolates, including a lactose operon and a trimethoprim resistance gene (labelled dhfr_2 in Figure 4, identical to *dfrG* using BLAST). Fifty-two genes were significantly associated with livestock isolates, including a copper resistance operon (containing *tcrB*) predominantly found in pigs and a streptogramin resistance gene (*vatD*) found exclusively in turkeys (26% of turkeys) (Fig. 4 and Table S4).

10.1128/mBio.01780-18.6 TABLE S4 Genes significantly associated with livestock or humans based on Scoary. Download Table S4, XLSX file, 0.04 MB. Copyright © 2018 Gouliouris et al. 2018 Gouliouris et al. This content is distributed under the terms of the Creative Commons Attribution 4.0 International license.

### Antibiotic resistance genes in E. faecium isolates from human and nonhuman sources.

**FIG 5:**
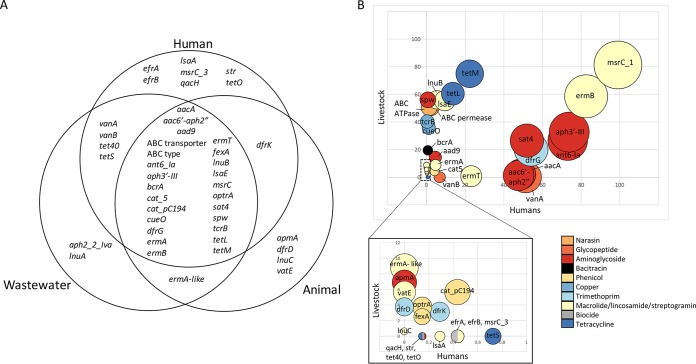
Genetic analysis of antibiotic resistance genes in E. faecium. (A) Venn diagram showing antibiotic resistance genes identified in each reservoir. (B) Bubble graph showing the prevalence of genes in isolates from humans versus animals. Lower graph shows an expanded view of low-prevalence genes from the upper graph. Size of the bubble represents the number of isolates that the gene was identified in. Bubbles are colored by antibiotic class.

We then undertook an analysis of accessory genes encoding antibiotic resistance across the 1,144 deduplicated isolates in the collection. Twenty-six out of 44 genes were identified in both human and livestock isolates (Fig. 5A). In many cases, these predominated in one reservoir or the other, but there were notable examples where genes were frequent in both, including macrolide and aminoglycoside, and, to a lesser extent, tetracycline resistance genes, likely reflecting the prominent roles these antibiotics have in both human and veterinary medicine (Fig. 5B; note that the proportions have the limitation that the sampling strategy was different for different reservoirs). Strikingly, several genes conferring resistance to antibiotics or metals used predominantly or exclusively in animals were identified at low levels in human invasive isolates (0.7%, 0.4%, and 1.1% carried *bcrA* [encoding bacitracin resistance], *tcrB*, and *cueO* [encoding copper resistance], respectively). The human isolates harboring these genes belonged to clade A1 (*n* = 6), clade B (*n* = 4), and BAPS5 (*n* = 2). Two genes coding for an ABC-type transporter associated with resistance to the antiprotozoal agent narasin, used in poultry (27), were common in chicken and turkey isolates (95%) and found at a very low level in human invasive isolates (1.4% to 2.3%; predominantly from clade A1 [90% to 94%]). Additionally, we identified *optrA*, a gene associated with linezolid and phenicol resistance (28), in one human isolate (clade A1) and six pig isolates (BAPS5, *n* = 5; clade A1, *n* = 1). *optrA* was also detected in one isolate from wastewater.

**FIG 6:**
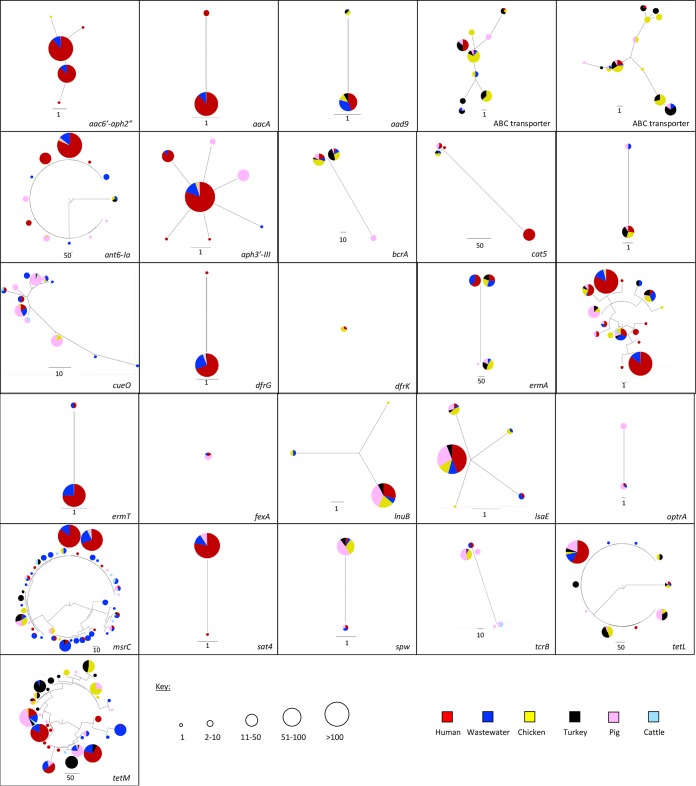
Comparison of antibiotic resistance gene variants in E. faecium isolates from humans, livestock and wastewater. Each figure section shows a phylogenetic tree of an antibiotic resistance gene based on SNPs. Pie charts show the source of the isolates in which each gene variant was found (see key for colors), and the size of the pie chart indicates the number of isolates that variant was identified in (see key).

Next, we evaluated the degree to which the genes from the two sources were related by constructing phylogenetic trees for each antibiotic resistance gene (Fig. 6). All 26 genes had evidence of gene sharing between human and livestock isolates based on 100% gene sequence identity, although the proportion of isolates involved was highly variable. The distribution of thirteen genes conferring resistance to antibiotics that are used in livestock and also important for human health (29) is shown in Table S5 (note that the proportions have the limitation of the sampling strategy). In most cases, the proportion of human and livestock isolates that shared genes was very low. Exceptions included *aph3*′-*III* (present in 69.1% of human and 19.5% livestock isolates) and *tetL* (present in 13.3% of human and 28.3% livestock isolates).

10.1128/mBio.01780-18.7 TABLE S5 Table of the thirteen genes that confer resistance to antibiotics used in livestock and that are important for human health. Download Table S5, XLSX file, 0.04 MB. Copyright © 2018 Gouliouris et al. 2018 Gouliouris et al. This content is distributed under the terms of the Creative Commons Attribution 4.0 International license.

Two resistance genes (encoding resistance to the aminoglycoside-related antibiotic spectinomycin [*spw*] and to tetracycline [*tetM*]) were further evaluated to determine whether sharing between reservoirs was associated with a specific mobile element (Table S6). These genes were selected because they were present in a high proportion of livestock isolates and their prevalence in livestock exceeded that in humans. Two variants of *spw* were shared by one human/one livestock and four human/88 livestock isolates, respectively. The gene was located in a 26.2-kb or 9.9-kb to 12.4-kb region shared by human and livestock isolates (0 to 1 SNPs different, equating to less than two months of evolution based on the seven SNP/core genome/year cutoff) in 1/1 and 3/4 human isolates, respectively (Fig. S2). In the remaining human isolate, the gene was located in a shared 3-kb region located adjacent to the contig end, which contained a gene encoding a hypothetical protein, *spw*, *apt*, and *aadE2*. Five variants of *tetM* were shared, three of which were at a low prevalence (<3.1%) in both reservoirs and not analyzed further. The remaining two variants were shared by one human and 16 livestock and by 11 and 38 livestock isolates, respectively. The gene was located in 6.7- to 21.3-kb regions shared by human and livestock isolates (0 to 1 SNPs difference), which contained one or more conjugative transposon genes in 1/1 and 10/11 human isolates, respectively (Fig. S2).

10.1128/mBio.01780-18.2 FIG S2 Regions of sequence containing the *spw* (A) or *tetM* (B) gene in human isolates, which are shared with one or more livestock isolates. Download FIG S2, PDF file, 0.1 MB. Copyright © 2018 Gouliouris et al. 2018 Gouliouris et al. This content is distributed under the terms of the Creative Commons Attribution 4.0 International license.

10.1128/mBio.01780-18.8 TABLE S6 Genetic context of the antibiotic resistance genes *spw* and *tetM*. Download Table S6, XLSX file, 0.04 MB. Copyright © 2018 Gouliouris et al. 2018 Gouliouris et al. This content is distributed under the terms of the Creative Commons Attribution 4.0 International license.

Finally, we determined the genetic context of the vancomycin resistance genes in the three meat VREfm isolates and compared it to that of the non-deduplicated full isolate collection (*n* = 1,442). The whole *vanA* Tn*1546* transposon was identified in single contigs of between 14.6 and 29.9 kb in the three meat isolates, which contrasts with the *vanA* transposons from the human isolates, which were often present on multiple contigs due to interruption by IS elements. In all three meat *vanA* contigs, the Tn*1546* transposon was flanked by a truncated streptomycin resistance *str* gene, and the largest contig also contained an *ermB* gene. The contig sequences matched >99% to pVEF4 (accession number FN424376), a plasmid isolated from a Norwegian poultry farm previously exposed to avoparcin (30). The isolate with the best match to pVEF4 was sequenced using PacBio technology, and a circularized plasmid of 45.7 kb (termed VREN1260_c3 here) was found to be highly similar to pVEF4 (99% identity over 96% of the sequence). Mapping reads of the whole collection against VREN1260_c3 showed that the other two meat isolates covered >97.9% of the sequence, while isolates from other reservoirs covered between 12.8% to 71.5%, suggesting that this plasmid has not spread to human invasive VREfm isolates in the UK. Importantly, in the human isolates there was no evidence of reads mapping to the sequences immediately upstream or downstream of the *vanA* transposon, suggesting that the *vanA* element was not shared between the human and meat isolates as part of a smaller transposable element. All three meat isolates also mapped to the ABC-type transporter genes present in VREN1260_c3, associated with narasin resistance, in addition to the *ermB* gene.

## DISCUSSION

This represents the first survey of VREfm conducted in British livestock since 2003, when a study in England and Wales reported the isolation of VREfm from 27/33 poultry and 4/14 pig farms (10). No VREfm was isolated from livestock in this study, suggesting that the rates of VREfm in livestock have fallen since the last survey in 2003. This is likely to be related to the ban in 1997 on the use of avoparcin as a growth promoter (7), 3 years after the first description of VREfm in British livestock (31).

One possible route of E. faecium and antibiotic resistance gene transfer from livestock to humans is through the food chain via meat. Surveys of retail meat drawn from producers over a wide geographic region demonstrated that low-level exposure to VREfm in 1% to 2% of uncooked meat products (3.7% of chicken meat) represents an ongoing potential zoonotic risk. All three products positive for VREfm were from the United Kingdom, and the E. faecium strains belonged to an animal-associated lineage, suggesting that VREfm has not been eradicated entirely from livestock, similar to the experience of other countries. However, the low frequency, combined with the bactericidal effect of heat during cooking, means that the risk of human exposure to VREfm in cooked food is likely to be very low. Indeed, we found no evidence that the *vanA* transposon or plasmid from the meat isolates was shared with any of the human or wastewater VREfm isolates. Although the number of VREfm isolates from meat was small, recovery of the same strain from chicken meat from two different supermarket chains at two different time points is consistent with the presence of a specific lineage of VREfm associated with poultry. The *vanA* plasmid was highly homologous to a plasmid (pVEF4) isolated in Norwegian poultry in 1998, with a pVEF4-like plasmid also reported very recently from Danish chicken meat (32) suggesting its widespread geographic and temporal dissemination. Possible reasons for persistence of vancomycin resistance genes 18 years after the ban of avoparcin in this plasmid include coselection by other antimicrobials used in livestock, such as macrolides or narasin, as suggested by the presence of *ermB* and ABC-type transporter genes (27, 33), and presence of a toxin-antitoxin system. Other possibilities include persistence in the farm or abattoir environment, with carryover of VREfm between successive flocks due to environmental survival and inadequate cleaning (10, 34), or possibly through avian carriage and transfer. Overall, our findings support the continued ban of avoparcin to limit VREfm spread within the livestock population and the potential spillover into human community carriage. While we cannot exclude the possibility of historical transfer of vancomycin resistance genes between animals and humans, we demonstrate that livestock is unlikely to be playing a significant role in the persistence of vancomycin resistance genes in human invasive isolates in the United Kingdom, where the situation now resembles that of the United States, where avoparcin was never used in agriculture.

We found largely host-adapted populations of E. faecium using phylogenetic analysis of the core genome, with the majority of livestock isolates from the United Kingdom located in lineages that were distinct from the hospital-associated clade. Despite this, there was evidence of limited overlap between isolates of livestock and hospital origin. Isolates causing human infection were found in the livestock-associated clade A2, as previously reported (21), although they were not closely related (minimum distance, 129 SNPs). Surprisingly, some livestock isolates resided in the previously considered human commensal clade B, and, more importantly, pig isolates resided in the hospital-associated clade A1. Previous genomic evidence of livestock isolates in this clade is lacking and limited to older typing techniques, which detected ST18 in the pig environment (24, 25). Here, porcine E. faecium (ST17, ST18, ST262, and ST947) was related to human infection isolates from 2001 to 2004, indicating potential human-to-pig or pig-to-human transmission more than a decade ago. The pig isolates were predominantly ampicillin resistant and had typical adaptations to the porcine environment, such as copper resistance genes.

Further evidence for distinct populations was provided by two different comparisons of the accessory genome, findings from which were consistent with a divergent evolutionary history and host adaptation for human- and animal-derived isolates. These analyses identified overrepresentation of a copper resistance operon in isolates from pigs and an exclusive association between a streptogramin resistance gene (*vatD*) and turkey isolates, which is consistent with the use of copper as a growth promoter in pigs (35) and historical use of virginiamycin as a growth promoter in poultry until its ban in 1998 (36).

Our analysis of mobile genes that encode resistance to antibiotics used in humans and livestock demonstrated that 26 different genes were present in human and livestock isolates. This indicates that such genes are ubiquitous but does not distinguish between frequent human-animal exchange and independent, parallel acquisition of resistance in the two groups. In-depth analysis of resistance genes to thirteen antibiotics used in humans and animals showed that the proportion of human and livestock isolates that shared the same gene was very low. Furthermore, evaluation of resistance to agents used predominantly or exclusively in animals (such as bacitracin or narasin and copper, respectively) demonstrated that such genes were present at low levels in human invasive isolates. This would argue for limited exchange of resistance genes between the two groups or, alternatively, lack of persistence due to absence of selective pressure. Important exceptions were the presence of genetically identical genes in human- and animal-derived isolates that encoded resistance to antibiotics commonly used in both human and veterinary medicine, such as tetracyclines, macrolides, and aminoglycosides. This is of concern, as aminoglycosides and macrolides are included in the WHO list of critically important antibiotics and tetracyclines in the list of highly important antibiotics (37).

Finally, we identified closely related isolates from farms of the same livestock species (with the exception of cattle) and from chickens and turkeys. While the farms were not colocated, one possible explanation is that chicken, turkey, and pig farms each belonged to the same company. Additionally, pigs from the different farms were likely to have shared a great-great-grandmother, with the potential for vertical transmission. In contrast, the cattle farms, which did not share strains, were owned by independent farmers. This suggests that the E. faecium population in livestock is highly mobile between farms, a finding of note for farmers and veterinary agencies.

A limitation of our study is the lack of human carriage isolates. Municipal wastewater is considered to represent pooled human gut bacterial populations (38). We found that both human- and animal-derived E. faecium isolates were represented in samples taken from wastewater plants, with farm and wastewater isolates separated by as few as 8 SNPs. There was no known discharge of farm effluent into the wastewater plants at the time of sampling, which suggests that clade A2 (animal-associated) isolates may be carried by healthy humans. Human carriage studies will be required to provide more conclusive evidence.

In conclusion, this study represents the first large-scale whole-genome sequencing study investigating the relationship between livestock and human infections. The results of this study confirm previous findings (21, 22) that E. faecium strains from livestock are not the cause of significant human disease but provides evidence for limited sharing of hospital-associated strains with pigs and of resistance genes with livestock species. Further studies are needed to determine whether our findings are relevant to different settings globally, where proximity to livestock and animal husbandry differ.

## MATERIALS AND METHODS

### Ethical approvals.

Approval to obtain and sequence isolates from patients with bloodstream infection in the United Kingdom and Ireland was obtained from the National Research Ethics Service (reference 12/EE/0439) and the CUH Research and Development (R&D) Department (reference A092685). Approval for bacterial isolation from wastewater and farms was obtained from the National Research Ethics Service (reference 14/EE/1123), the Cambridge University Hospitals NHS Foundation Trust R&D Department (reference A093285), and the Department of Veterinary Medicine, University of Cambridge (study CR112).

### Isolation of E. faecium strains from livestock, retail meat, and wastewater.

A cross-sectional survey was performed between August 2014 and April 2015 to isolate E. faecium strains from 20 livestock farms (ten cattle and ten pig) in the East of England. The farms were chosen randomly from a pool of farms served by veterinary surgeons at the University of Cambridge. Farm characteristics are shown in Fig. S1C. Poultry reared at nine farms (four chicken and five turkey) in the East of England were sampled at two abattoirs between February and April 2015. Farm details, sampling strategy, and processing of samples are described in the Supplemental Methods. All samples were processed on the day of collection and subjected to direct and enriched cultures.

Two cross-sectional surveys were performed between February and April 2015 to detect vancomycin-resistant E. faecium isolates from retail meat purchased from high-street supermarkets. In the first survey, 103 prepackaged meat samples (52 pork and 51 chicken), all of UK farm origin, were purchased from five locations in the United Kingdom, including Cambridge (2 to 11 supermarket brands sampled per location) and frozen at −20°C immediately after purchase. In the second survey, 97 products (16 beef, 30 chicken, 42 pork, 7 turkey, 1 pork/beef, and 1 venison) from multiple countries of origin were obtained from 11 major supermarkets in the Cambridge area and tested within 24 h of purchase. Sample processing is described in detail in the Supplemental Methods.

Collection of wastewater from 20 municipal wastewater treatment plants across the East of England between June 2014 and January 2015 has been described previously (39). In brief, ten plants were situated downstream of acute NHS Hospital Trusts, and ten plants were not connected to acute hospital effluent. Paired samples of untreated and treated wastewater were obtained from each plant. Sampling strategy and sample processing are described in the Supplemental Methods.

### E. faecium isolates associated with bloodstream infection.

A total of 793 E. faecium genome sequences reported previously (18, 23) were downloaded from the Wellcome Sanger Institute. Of these, 308 isolates were isolated at the Cambridge University Hospitals NHS Foundation Trust (CUH) diagnostic laboratory, Cambridge (UK), and included the first available isolate from all consecutive patients with either vancomycin-resistant (*n* = 200) or vancomycin-susceptible (*n* = 93) E. faecium bloodstream infections between 2006 and 2012 (18). To provide national context, we included 474 isolates (roughly equal numbers of VREfm and vancomycin-susceptible Enterococcus faecium [VSEfm]) from the British Society for Antimicrobial Chemotherapy (BSAC) Bacteraemia Resistance Surveillance Programme (www.bsacsurv.org) between 2001 to 2011 from 40 hospital diagnostic laboratories across the United Kingdom and Ireland (23), and 11 isolates from the National Collection of Type Cultures (NCTC). Phenotypic susceptibility to vancomycin was determined previously using the BSAC disc diffusion method (40) or the VITEK2 system (bioMérieux, Marcy l’Etoile, France) with the AST-P607 card (18, 23). This demonstrated that 445/793 isolates (56%) were resistant to vancomycin.

### Sequencing and analysis of sequence data.

DNA libraries were prepared according to the Illumina protocol and sequenced on an Illumina HiSeq 2000 with 100-cycle paired-end runs. The new sequence data generated by this study have been deposited in the European Nucleotide Archive (www.ebi.ac.uk/ena) under the accession numbers provided in Table S2. All bacterial genomes used in the analysis (782 human bloodstream, 11 NCTC, 383 wastewater, 253 livestock, and three meat) were assembled using Velvet and annotated using Prokka. Multilocus sequence typing (MLST) was determined from genome assemblies using MLST Check (41), with novel alleles and STs deposited in the pubMLST database (https://pubmlst.org/efaecium/). Absence of the *pstS* gene in two isolates was confirmed by a second method based on read mapping (Short Read Sequence Typing for Bacterial Pathogens [SRST2]) (42). Pangenomes were estimated for the livestock and meat isolates alone and for the entire study collection plus ten reference genomes using Roary, with a 90% identity (ID) cutoff and genes classed as “core” if they were present in 99% of isolates. Maximum-likelihood trees were created using RAxML, based on SNPs in the core genes. The livestock and meat phylogeny was based on 99,377 SNPs in 1,523 core genes (total of 12,393 genes); the livestock, meat, clinical, and wastewater phylogeny was based on 95,115 SNPs in 1,166 core genes (total of 22,284 genes).

Isolates were clustered using Bayesian analysis of population structure (BAPS) (43) based on the core genes of the study collection and reference genomes, described in more detail in the Supplemental Methods. The resulting BAPS groups are clusters of related isolates based on Bayesian inference of the genetic structure of the population. The study collection was deduplicated for further analysis by removing all isolates that were 0 to 2 SNPs from another isolate from the same location (same farm, wastewater treatment plant, or hospital) based on SNPs in the core genes. All subsequent analyses were performed on this deduplicated data set. For analysis within BAPS groups, clade A isolates were mapped to a reference genome from the same BAPS group using SMALT, described further in the Supplemental Methods. SNPs were identified within each BAPS group (or second-order BAPS groups for BAPS4), and pairwise SNP differences between isolates were identified using an in-house script. A network file for the livestock and wastewater isolates was created in R with cutoffs of seven and 35 SNPs used to define relationships, and plotted in MicroReact. Principal component analysis was performed across the whole deduplicated collection based on the accessory genes from Roary using R. Scoary (44) was used to determine genes associated with specific reservoirs and was performed across the livestock and human invasive collection. The presence of antibiotic, biocide, and metal resistance genes was determined using Ariba with a 90% ID cutoff (45). Sequences of genes classed as present in Ariba were extracted from the Ariba output. Further details are provided in the Supplemental Methods. Statistical analysis was performed using Fisher’s exact test with a *P* value of <0.05.

DNA from one VREfm meat isolate was extracted using the phenol-chloroform method, sequenced using the PacBio RS II instrument, and sequence data processed as previously described (18). Genomic reads were mapped against this plasmid using SMALT, as above, and visualized using an in-house tool. The sequence for this isolate is available in the European Nucleotide Archive under accession number ERS1420638, with the plasmid containing *vanA* being contig 3.

### Data availability.

Accession numbers for the sequence data generated by this study are provided in Table S2, with sequences deposited in the European Nucleotide Archive (www.ebi.ac.uk/ena).

10.1128/mBio.01780-18.2 TEXT S1 Supplemental methods. Download TEXT S1, DOCX file, 0.03 MB. Copyright © 2018 Gouliouris et al. 2018 Gouliouris et al. This content is distributed under the terms of the Creative Commons Attribution 4.0 International license.
