# Supplementary material for: Genomic Surveillance of Enterococcus faecium Reveals Limited Sharing of Strains and Resistance Genes between Livestock and Humans in the United Kingdom
Source: mBio. 2018 Nov 6;9(6):e01780-18. doi: 10.1128/mBio.01780-18 (PMC6222123; doi:10.1128/mBio.01780-18)
Supplement: FIG S1 [file mbo005184139sf1.pdf]

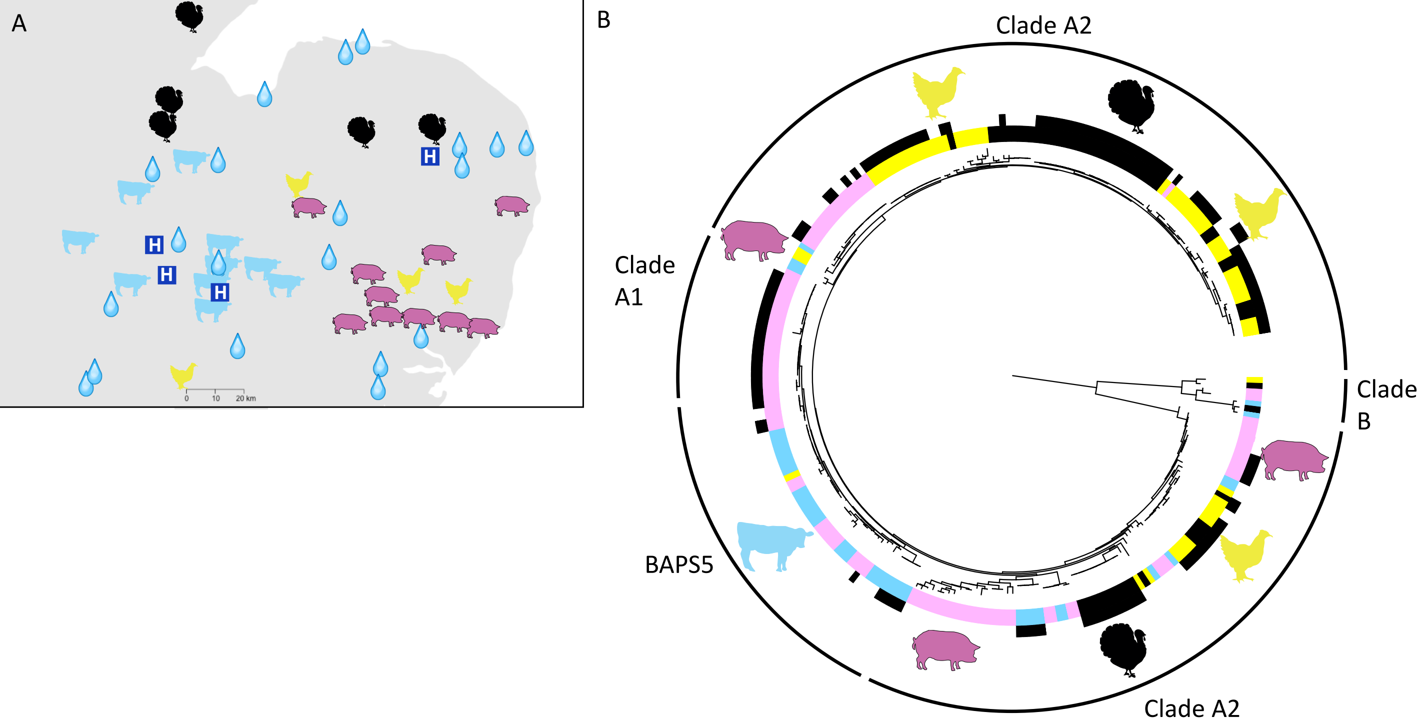

C

Farm number	Livestock species	Indoor	High-intensity	Number of animals	Number of sheds	Number of samples taken	*E. faecium* positive	Ampicillin-resistant *E. faecium* positive	Vancomycin-resistant *E. faecium* positive	Number of isolates sequenced
1	Dairy cattle	Yes	No	220	4	5	Yes	No	No	5
2	Pig	Yes	Yes	750	1	4	Yes	Yes	No	14
3	Pig	Yes	Yes	450	2	7	Yes	No	No	5
4	Pig	Yes	Yes	2600	3	4	Yes	Yes	No	12
5	Pig	Yes	Yes	1400	2	4	Yes	Yes	No	12
6	Pig	Yes	Yes	480	2	4	Yes	Yes	No	13
7	Pig	Yes	Yes	1124	1	5	Yes	No	No	5
8	Pig	Yes	Yes	1683	4	6	Yes	Yes	No	7
9	Pig	Yes	Yes	841	1	4	Yes	Yes	No	13
10	Pig	Yes	Yes	1770	9	9	Yes	Yes	No	7
11	Pig	Yes	Yes	1600	4	8	Yes	Yes	No	7
12	Beef cattle	No	No	37	1	4	Yes	No	No	5
13	Beef cattle	No	No	200	3	5	Yes	Yes	No	9
14	Beef cattle	No	No	30	1	1	Yes	No	No	1
15	Beef cattle	No	No	28	2	4	Yes	No	No	2
16	Dairy cattle	Yes	No	560	1	4	Yes	No	No	2
17	Dairy cattle	Yes	Organic	284	1	5	Yes	Yes	No	9
18	Dairy cattle	Yes	No	137	1	2	Yes	No	No	4
19	Chicken	Yes	Yes	26400	Not available	6	Yes	Yes	No	15
20	Chicken	Yes	Yes	19971	Not available	7	Yes	Yes	No	15
21	Turkey	Yes	Yes	1584	Not available	5	Yes	Yes	No	14
22	Turkey	Yes	Yes	792	Not available	4	Yes	Yes	No	10
23	Turkey	Yes	Yes	603	Not available	4	Yes	Yes	No	11
24	Turkey	Yes	Yes	792	Not available	4	Yes	Yes	No	13
25	Turkey	Yes	Yes	100	Not available	4	Yes	Yes	No	11
26	Beef cattle	No	No	234	5	4	Yes	No	No	5
27	Chicken	Yes	Yes	6288	Not available	8	Yes	Yes	No	14
28	Chicken	Yes	Yes	6600	Not available	8	Yes	Yes	No	13
29	Dairy cattle	Yes	No	176	1	3	No	No	No	0

Figure S1. Geographical and phylogenetic distribution of *E. faecium* isolates cultured from livestock, and farm characteristics. A: Map of the East of England region of the UK, showing the approximate locations of the farms (images indicate the animal species), wastewater treatment plants (indicated with water drops), and hospitals in the region (indicated by a white 'H' in a blue square). B: Maximum likelihood tree of the 256 livestock and meat isolates based on 99,377 SNPs in 1,523 core genes. Colored rings show the animal species (blue, cow; pink, pig; black, turkey; yellow, chicken), ampicillin resistance (black, resistant; white, susceptible/intermediate) and vancomycin resistance (black, resistant; white, susceptible) from inside to out. Outermost ring indicates the Clade designations. C: Table showing the farm characteristics, listed chronologically according to sampling date.
