# Supplementary material for: Genomic Surveillance of Enterococcus faecium Reveals Limited Sharing of Strains and Resistance Genes between Livestock and Humans in the United Kingdom
Source: mBio. 2018 Nov 6;9(6):e01780-18. doi: 10.1128/mBio.01780-18 (PMC6222123; doi:10.1128/mBio.01780-18)
Supplement: FIG S2 [file mbo005184139sf2.pdf]

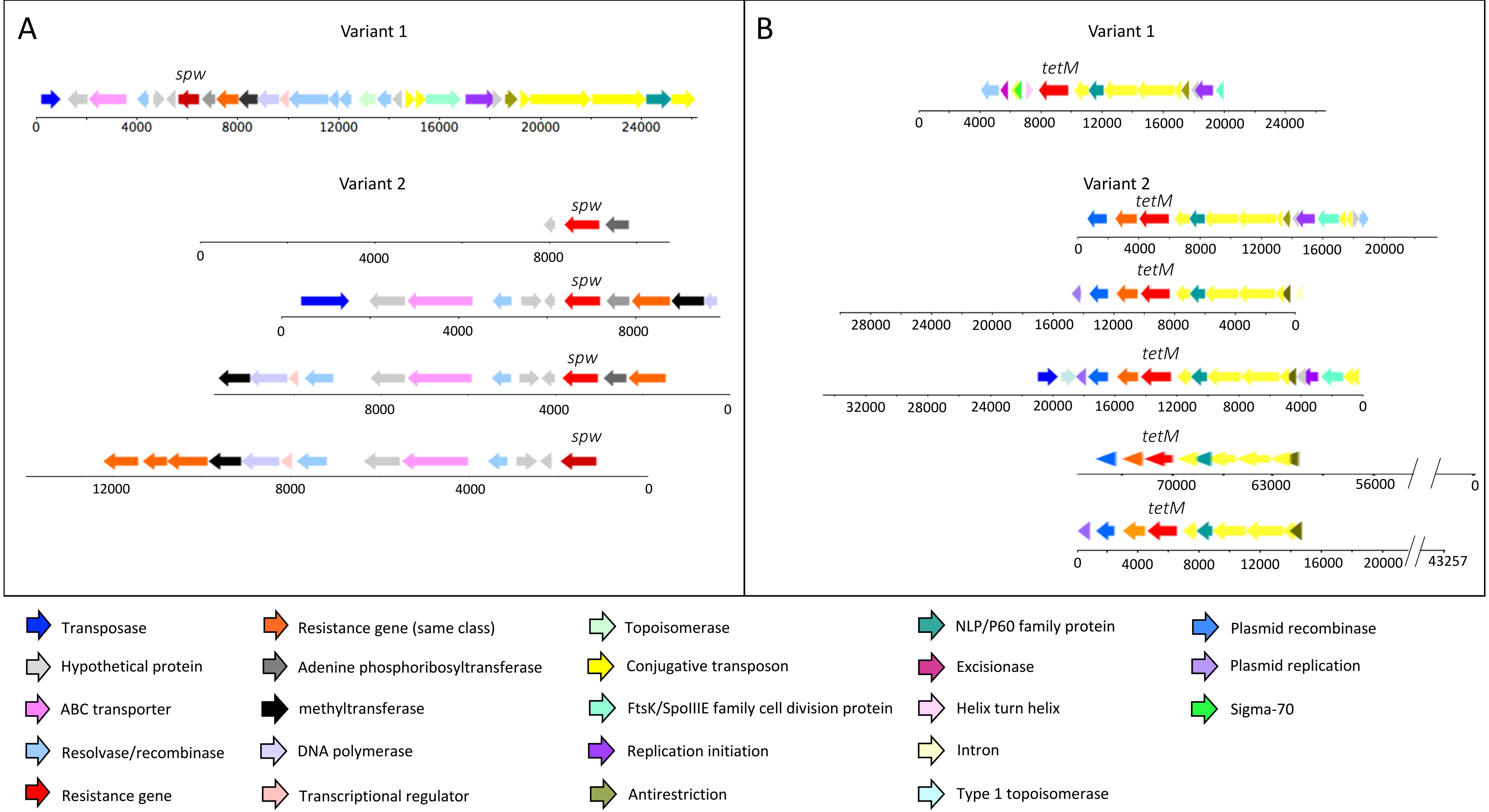

Figure S2. **Regions of sequence containing the *spw* (A) or *tetM* (B) gene in human isolates, which are shared with one or more livestock isolates.** A: Each line shows the length of the contig containing the *spw* gene in a human isolate (from top to bottom: 10678_8#18, 10678_8#61, 10678_8#75, 10733_3#48 and 10702_8#32). Genes that are in the region of sequence shared between the human and livestock isolates are indicated (colored arrows). Two variants of the *spw* gene based on nucleotide sequence are shown by Variant 1 (identified in 1 human and 1 livestock isolate) and Variant 2 (identified in 4 human and 88 livestock isolates). B: Each line shows the length of the contig containing the *tetM* gene in a human isolate (from top to bottom: 10733_5#50, 10678_8#75, 10702_8#66, 10733_1#10, 10733_8#78 and 10733_8#69). Genes that are in the region of sequence shared between the human and livestock isolates are indicated (colored arrows). Only human isolates with shared regions greater than 10kb in length are shown. Two variants of the *tetM* gene based on nucleotide sequence are shown by Variant 1 (identified in 1 human and 16 livestock isolates) and Variant 2 (identified in 11 human and 38 livestock isolates)
