# Supplementary material for: Genomic Surveillance of Enterococcus faecium Reveals Limited Sharing of Strains and Resistance Genes between Livestock and Humans in the United Kingdom
Source: mBio. 2018 Nov 6;9(6):e01780-18. doi: 10.1128/mBio.01780-18 (PMC6222123; doi:10.1128/mBio.01780-18)
Supplement: TEXT S1 [file mbo005184139s1.docx]

**Supplementary Methods**

**Isolation of *E. faecium* from farms**

Beef cattle farms were outdoor units and contained 28 to 234 animals, while dairy cattle farms were indoor units with 137-560 animals. Pig farms were growing (n=9) and breeding (n=1) high-intensity indoor facilities housing from 450 to 2,600 pigs and employed an all-in all-out production policy for infection control and high biosecurity standards. The area covered and the number of possible samples that could be processed were maximized by taking pooled samples, in which up to 50 aliquots each with a total weight of around 50 g of freshly passed fecal material were collected from each major area in a given farm (for example, a pen) using a sterile scoop (Thermo Scientific Sterilin™ Polystyrene Universal Containers, Thermo Fisher Scientific, Paisley, UK). Each pool was collected into dry sterile 150 mL containers (Thermo Scientific Sterilin™ Polystyrene Containers). A median of four pooled samples (range 1-5) were taken from each cattle farm, and a median of 4.5 samples (range 3-9) taken from each pig farm, resulting in a total of 85 pooled samples (34 cattle and 51 pig). In addition, cecal contents were collected from two deceased pigs on the same farm at the time of necropsy.

Both poultry abattoirs had a high-throughput, each processing in excess of 100,000 birds daily. Two sample types were taken from animals from each farm: (i) pooled feces with a total weight of around 50 g from 10 - 20 transportation crates immediately after the animals were removed; (ii) pools of cecal material from up to ten birds after slaughter. Each sample was taken using a sterile scoop and a sterile surgical scalpel was used for cecal dissection. A median of 4 (range 2-4) cecal pools and a median of 4 (range 3-4) fecal pools were collected from animals from each chicken farm. A median of 1.5 (range 1-2) cecal pools and a median of 2.5 (range 2-3) fecal pools were collected from animals from each turkey farm. This resulted in a total of 49 pooled samples (29 chicken and 20 turkey).

All samples were processed on the day of collection. Pooled fecal samples were diluted 1:1 with sterile phosphate-buffered saline (Cell Signaling Technology, Hitchin, UK), mixed vigorously, and subjected to direct and enriched cultures. For direct cultures, 100 μL of each homogenized sample was plated onto the selective media described in the wastewater section and incubated for 48 hours at 37°C in air. For enrichment cultures, 1 mL of each sample was added to 9 mL of selective broth and incubated for 24 hours in a shaking incubator at 37°C. Selective broths consisted of BBL^TM^ Enterococcosel^TM^ broth (BD, Oxford, UK) with or without antibiotic supplementation (30 mg/L ampicillin or 6 mg/L vancomycin). Broths that turned black as a result of esculin hydrolysis were sub-cultured onto the corresponding selective agar plates and incubated as described above.

**Isolation of *E. faecium* from meat**

The origin of retail meat samples was as follows: beef and turkey originated from the UK and Ireland; pork from the UK and Europe; chicken from the UK, Poland, Ireland, Hungary, Germany, Brazil, and Thailand; and venison from New Zealand. Samples from both surveys were processed at the Department of Veterinary Medicine, University of Cambridge. Frozen samples were thawed, and the exterior packaging was thoroughly disinfected with alcohol. A 5g sample of meat was excised aseptically, mixed with 45 mL of peptone broth and homogenized in a Stomacher® paddle blender (Stomacher® 80 Biomaster, Seward, Worthing, UK) for two minutes. In addition, swabs were obtained from whole chicken carcasses and incubated in 3 mL brain heart infusion (BHI) broth (FloqSwabs, Copan Italia spa, Brescia, Italy). Following aerobic incubation of enrichment broths at 37°C for 24 hours, 100 μL was inoculated onto *Brilliance*™ VRE chromogenic media, which were processed as described above.

**Isolation of *E. faecium* from wastewater treatment plants**

One mL of triplicate serial ten-fold dilutions, 10 mL of treated and untreated wastewater and 100 mL of treated wastewater samples were concentrated using the filtration technique onto 0.45 μm pore size, mixed cellulose ester filter membranes (S-Pak, Merck Millipore, Darmstadt, Germany). Membranes were then placed onto the surface of a range of selective plates representing increasing levels of antibiotic selective pressure and incubated for 48 hours at 37°C in air. The plates consisted of Slanetz-Bartley (SB) agar (Oxoid, Basingstoke, UK), which enriches for enterococci but does not select for antibiotic resistance; enterococci-selective medium BBL^TM^ Enterococcosel^TM^ agar (BD, Oxford, UK) supplemented with 30 mg/L ampicillin (Sigma-Aldrich, Poole, UK); and vancomycin-resistant enterococci (VRE)-selective medium *Brilliance*^TM^ VRE chromogenic agar (Oxoid, Basingstoke, UK). At least one isolate of each bacterial colony morphology type suspected to be *Enterococcus* sp. was picked and speciated using matrix-assisted laser desorption/ionization time-of-flight mass spectrometry (MALDI-TOF MS; Biotyper version 3.1, Bruker Daltonics, Coventry, UK). Antimicrobial susceptibility testing was determined using the AST-P607 card on the VITEK^®^ system (bioMérieux, Marcy l’Étoile, France) calibrated against EUCAST breakpoints (<http://www.eucast.org/clinical_breakpoints/>).

**BAPS analysis**

Isolates were clustered using Bayesian Analysis of Population Structure (BAPS) (1) based on the core genes of the study collection and reference genomes, with private SNPs excluded before the analysis (17,834 private SNPs were excluded out of a total of 95,115 SNPs). hierBAPS software was run with five replicates of the posterior maximization algorithm using prior upper bounds of 200-500 clusters. All runs resulted in the same maximum *a posteriori* partition estimate, indicating high peakedness of the posterior distribution over the clustering space. For analysis within BAPS groups, isolates were mapped to a reference genome from the same BAPS group using SMALT. Where a reference genome was not available, the *de novo* assembly of the study isolate with the lowest number of contigs was used as the reference. Mobile genetic elements (MGEs) and recombination were removed as described previously (2) in addition to removing IS elements identified by ISfinder (www-is.biotoul.fr). The diversity in BAPS4 was too high to reliably identify recombination events, so the cluster was further sub-divided into second order BAPS clusters and MGEs and recombination removed as above.

**Principal component and Scoary**

For the principal component analysis, spearman rho correlations were calculated using Past3. The 100 genes with the highest scoring spearman rho correlation with BAPS9 and BAPS6, respectively, were considered. The inputs for Scoary were the results of Roary with paralogs removed, and a RAxML tree based on SNPs in the core genes. Gene associations with vancomycin resistance and high-level gentamicin resistance were used as validation datasets. Genes were counted as significant if the Best and Worst pairwise values were p<0·05 and the Bonferroni p-value was <0·05.

**Antimicrobial resistance determinants**

The ResFinder database (compiled in 2012), which had been manually curated, was used as the input database. A literature review was conducted to identify antibiotic resistance determinants absent from the ResFinder database and sequences for 22 genes were added (gene name, accession number, gene position): *aadA*, AF052459.1, 107-898bp; *aadA2*, AB253625.1, 79-858bp; ABC transporter permease protein, CP010059.1, 782341-783954bp; ABC type multidrug transport system ATPase, CP010059.1, 781436-782317bp; *bcrA1*, AY496968, 2325-3251bp; *bcrA2*, KJ645709.1, 20875-21792bp; *cadA*, J04551.1, 1064-3247bp; *copB*, DQ089807.2, 11993-14071bp; *cueO*, HM565232.1, 5794-7377bp; *efrA*, AE016830.1, 2798453-2800180bp; *efrB*, AE016830.1, 2796687-2798456bp; *ermA*-like Tn554 variant, KT862775, 11897-12628bp; *ermB1* variant, ERR369948, 2737374..2738111bp; *lsaE*, KF421157.1, 6361-7845bp; *mco* p5578, CP001603.1, 2032-3192bp; *mco* pBMB171, CP001904.1, 266489-268153bp; *mcr1*, KP347127.1, 22413-24038bp; *msrC_3*, AE016830.1, 1392319-1393767bp; *optrA*, KP399637.1, 31477-33444bp; *sat4*, AF330699.1, 927-1469bp; *spw*, AHWI01000032.1, 1153-1962bp; *tcrB*, AY048044.2, 4747-6879bp. Genes were counted as present if Ariba identified them as ‘yes’ or ‘yes_non-unique’. Genes identified as ‘fragmented’ by Ariba were investigated further by performing a BLAST analysis for the gene of interest against the assembly of the isolate. If the BLAST match was 100% length and >90% ID then the gene was classed as present. We additionally ran an in-house script (https://github.com/simonrharris/map_resistome) to identify antibiotic resistance genes using the same input database and found that variants of *tetM* and *ermB* were not correctly identified by Ariba. The variants of these two genes were defined using BLAST against the assembled sequences with a cut-off of 100% length and identification of the highest percentage match(es).

Each gene alignment was manually assessed in SeaView, and sequences that were not of 100% length were removed from the analysis. For the remaining sequences, identical sequences were grouped. For *ermB*, *tetM,* and genes identified as ‘fragmented’ by Ariba, the sequences were extracted using BLAST and, where applicable, concatenated to the aligned sequences from Ariba. A single representative sequence for each group was used to create a phylogenetic tree for each antibiotic resistance gene using FastTree. The plasmid context of the antibiotic resistance genes was investigated by performing a BLAST comparison between contigs containing the gene of interest from the source category (human/livestock) against all contigs across the study collection. *spw* and *tetM* were selected for analysis as they were the most prevalent in livestock for two antibiotic classes of particular importance in human health, and had low prevalence in humans (increasing the likelihood that directionality was from livestock to humans).

REFERENCES

1. Cheng L, Connor TR, Siren J, Aanensen DM, Corander J. 2013. Hierarchical and spatially explicit clustering of DNA sequences with BAPS software. Mol Biol Evol 30:1224–1228.

2. Raven KE, Gouliouris T, Brodrick H, Coll F, Brown NM, Reynolds R, Reuter S, Török ME, Parkhill J, Peacock SJ. 2017. Complex routes of nosocomial vancomycin-resistant *Enterococcus faecium* transmission revealed by genome sequencing. Clin Infect Dis 64:15–17.
